# SpeckleGAN: a generative adversarial network with an adaptive speckle layer to augment limited training data for ultrasound image processing

**DOI:** 10.1007/s11548-020-02203-1

**Published:** 2020-06-18

**Authors:** Lennart Bargsten, Alexander Schlaefer

**Affiliations:** Institute of Medical Technology and Intelligent Systems, Hamburg University of Technology, Hamburg, Germany

**Keywords:** Deep learning, Synthetic image generation, Theory-guided neural networks, Speckle noise, Small datasets, Image segmentation

## Abstract

**Purpose:**

In the field of medical image analysis, deep learning methods gained huge attention over the last years. This can be explained by their often improved performance compared to classic explicit algorithms. In order to work well, they need large amounts of annotated data for supervised learning, but these are often not available in the case of medical image data. One way to overcome this limitation is to generate synthetic training data, e.g., by performing simulations to artificially augment the dataset. However, simulations require domain knowledge and are limited by the complexity of the underlying physical model. Another method to perform data augmentation is the generation of images by means of neural networks.

**Methods:**

We developed a new algorithm for generation of synthetic medical images exhibiting speckle noise via generative adversarial networks (GANs). Key ingredient is a speckle layer, which can be incorporated into a neural network in order to add realistic and domain-dependent speckle. We call the resulting GAN architecture SpeckleGAN.

**Results:**

We compared our new approach to an equivalent GAN without speckle layer. SpeckleGAN was able to generate ultrasound images with very crisp speckle patterns in contrast to the baseline GAN, even for small datasets of 50 images. SpeckleGAN outperformed the baseline GAN by up to 165 % with respect to the Fréchet Inception distance. For artery layer and lumen segmentation, a performance improvement of up to 4 % was obtained for small datasets, when these were augmented with images by SpeckleGAN.

**Conclusion:**

SpeckleGAN facilitates the generation of realistic synthetic ultrasound images to augment small training sets for deep learning based image processing. Its application is not restricted to ultrasound images but could be used for every imaging methodology that produces images with speckle such as optical coherence tomography or radar.

## Introduction

Cardiovascular diseases like atherosclerosis are the leading cause of death globally [12]. A common methodology for assessing the severity and progress of plaque building in coronary arteries is intravascular ultrasound (IVUS) as it provides information regarding the vessel wall and the composition of plaques.

In recent years, finding diagnoses has been more and more supported by algorithms which provide additional information to the physician. In particular, powerful deep learning methods gained significant importance due to their superior performance compared to many explicit algorithms. Typical applications are detection and classification of diseases or segmentation of different tissues.

A drawback is the need of large annotated training datasets in order to get useful results. Annotations are usually made by trained experts to ensure high quality. This naturally leads to a lack of high-quality data. To overcome these limitations, data augmentation methods are commonly used [18]. In addition to applying random transformations to the data samples (which do not alter their labels), the generation of artificial training data is a possible way to enlarge the training set. One way to generate synthetic data is to run simulations. These often rely on rather simple models or require in-depth domain knowledge leading to results which are either of low quality or quite time consuming.

Another promising method to generate artificial data is by training generative adversarial networks (GANs) [6]. Nevertheless, GANs also need sufficient amounts of training data to reach satisfactory performances. Often they are trained with more than 10,000 images or even 100,000 images when dealing with rather diverse datasets [15]. To reduce the amount of needed data, theory-guided operations or modules may be integrated into the neural network architecture [11]. These arise from theoretical considerations or physical models which can replace parts of the network. In this way, the amount of model capacity which would be used to learn these physical concepts is free to learn other features. In addition, theory-based network modules serve to regularize the training process and can thus lead to improved performance.

We designed such a theory-guided network module to add speckle noise to network feature maps and integrated it into a GAN architecture, which we called SpeckleGAN. This enables us to generate realistic IVUS images with very few training examples, while keeping the overall network architecture simple. Furthermore, the size of resulting speckles can vary for a single image and is learned during the training process. Finally, we show how we can improve IVUS image segmentation performance by means of pre-training a neural network with synthetic images by SpeckleGAN if only very limited data are available. Our method thus enables the training of high-capacity neural networks with few data by simultaneously prevent overfitting.

## Material and methods

### Speckle layer

Speckle is an interference phenomenon in imaging systems and occurs if the mean distance between scatterers is smaller than the resolution cell defined by the imaging methodology [2]. The size of the resolution cell is determined mainly by the wavelength of the carrier (or excitation) signal. Another condition for the developing of speckle is the presence of independent random phases of the scattered waves at the point of observation, usually generated by surface roughness (optics) or inhomogeneous volumes like tissue (ultrasound). Interference of these signals leads to characteristic speckle patterns.

The algorithm for the speckle layer resembles the one found in the appendix of [8] and is based on the principles of Fourier optics explained in [7]. In Fourier optics, one takes advantage of the fact that under certain simplifications the propagation and diffraction of wave signals can be expressed as Fourier transformations. Although the process of speckle formation differs in ultrasound systems, the resulting effect on the gray values is similar and we illustrate the approach in the context of a simple optical system.

**Fig. 1:**
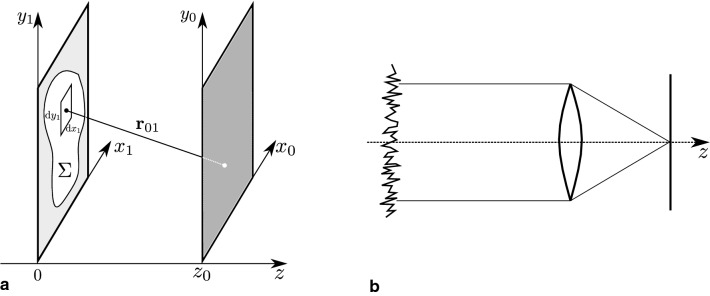
**a**: Sketch showing diffraction at an aperture. Variable naming corresponds to Eq. 1. **b**: Sketch of a simple imaging system with a rough object and a converging lens. Due to the roughness, the object’s signal exhibits a spatial distribution of random phases which leads to speckle patterns in the focal plane of the lens

The algorithm is based on an imaging system comprised of an illuminated rough object and a converging lens (see Fig. 1b). The propagation and focusing of the wave signal emitted by the object can be represented by two consecutive Fourier transformations. This is possible if some approximations are applied to the following general form of the diffraction integral. It describes how wave signals are diffracted at apertures and is defined as

1 $$\begin{aligned} U(x_0, y_0) = \frac{1}{j\lambda }\iint _{\Sigma }\frac{\exp {(jkr_{01})}}{r_{01}} \cos {(\mathbf {n}, \mathbf {r}_{01})}\; U(x_1,y_1)\,\text {d}x_1\,\text {d}y_1. \end{aligned}$$

Here, $U(x_0, y_0)$ denotes the field amplitude in the plane of observation, $U(x_1,y_1)$ the field amplitude in the aperture plane and $\Sigma$ the aperture. The vector $\mathbf {n}$ represents the normal of the aperture plane, *k* is the wave number, $\mathbf {r}_{01}$ the vector between a point on the aperture plane and another point on the plane of observation and $r_{01}$ its norm. See Fig. 1a for a corresponding sketch. Further details regarding the derivation of the formula and its application to the imaging system of Fig. 1b can be found in [7].

The speckle layer imitates the optical system of Fig. 1b and can be described by the following equation:

2 $$\begin{aligned} I_{sp}(x,y) = \left| \mathcal {F}^{-1}\left\{ \mathcal {F}\left\{ I(x,y)\cdot e^{j\,\varphi (x,y)}\right\} \cdot \text {rect}_d(x,y)\right\} \right| , \end{aligned}$$

where *I*(*x*, *y*) and $I_{sp}(x,y)$ denote the source and speckled image, respectively. $\mathcal {F}$ represents the Fourier transformation and $\text {rect}_d(x,y)$ the rectangular window function with edge length *d*. For the sake of simplicity we did not use a circular window function indicated by the lens in Fig. 1. On the one hand, we did not observe any difference in the visual appearance of the resulting speckle, on the other hand the calculation of a circular mask function is computationally more expensive, because the distance between every pixel to the image center has to be calculated in every training step. Equation 2 can be interpreted as a low-pass filter of the source image which is multiplied pixel-wise with random phases and is thus equivalent to

3 $$\begin{aligned} I_{sp}(x,y)&= \left| I(x,y)\cdot \text {e}^{j\,\varphi (x,y)}\,*\, \mathcal {F}^{-1}\left\{ \text {rect}_d(x,y)\right\} \right| \end{aligned}$$

4 $$\begin{aligned} I_{sp}(x,y)&= \left| I(x,y)\cdot \text {e}^{j\,\varphi (x,y)}\,*\,\text {sinc}_d(x,y)\right| . \end{aligned}$$

**Fig. 2:**
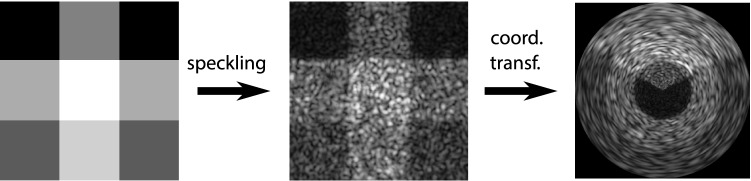
Exemplary speckle transformation of a test image with subsequent coordinate transformation in order to get warped speckles typical for IVUS images

Here, $*$ is the convolution operator and $\text {sinc}_d(x,y)$ the sinc-function with scale *d*. The edge length *d* of the rectangular window function defines the mean size of the resulting speckles and can be learned during training of the neural network. Smaller windows lead to larger speckle patches. We note that the runtime complexity of a convolution operation scales with $n^2$ while the fast Fourier transform (FFT) scales with $n\cdot \log (n)$. It is thus computationally more efficient to implement Eq. 2. In order to generate the typical speckle patterns for centric IVUS views, coordinate transforms from polar to Cartesian coordinates and vice-versa were added to the pipeline. An exemplary speckle transformation process is depicted in Fig. 2.

### SpeckleGAN architecture

To generate IVUS images with defined geometry regarding the artery lumen and the intima/media layers, a segmentation mask has to be used as a conditional input. A promising way to process the segmentation masks is by using spatially-adaptive normalization (SPADE) for semantic image synthesis [15]. SPADE layers transform segmentation masks (here, encoded as images with integer pixel values from {0, 1, 2}, where each value corresponds to a tissue class) into feature maps $\gamma$ and $\beta$ by feeding them through two convolutional layers, respectively. The segmentation masks are resized before feeding them into SPADE in order to have the same size as the feature maps which should be normalized. Pixel values $x^{in}_{n,c,h,w}$ of input feature maps to be normalized are transformed as follows:

$$\begin{aligned} x^{out}_{n,c,h,w} = \gamma _{c,h,w} \frac{x^{in}_{n,c,h,w} - \mu _c}{\sigma _c} + \beta _{c,h,w}, \end{aligned}$$

where the multi-index (*n*, *c*, *h*, *w*) refers to (sample in batch, channel, height, width). The parameters $\mu _c$ and $\sigma _c$ denote the channel-wise mean and standard deviation of $x^{in}_{:,c,:,:}$, respectively. A colon indexes the whole tensor dimension.

**Fig. 3:**
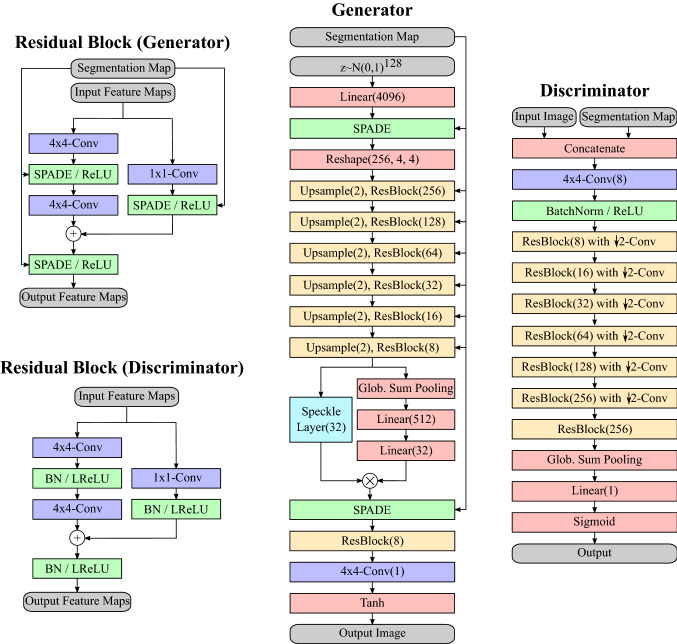
Sketch of the architecture of SpeckleGAN. Numbers in round brackets depict the respective numbers of output channels. Exceptions are *Upsample* (number depicts the scaling factor) and *Reshape* (number depicts the output’s channel and spatial dimensions)

Figure 3 gives an overview of the overall GAN architecture. Generator and discriminator consist of multiple residual blocks [9]. In the generator, SPADE [15] layers are used to condition the generated image to a given segmentation mask. The first convolutions in all SPADE layers have 64 output channels. Batch normalization precedes the affine transformation by SPADE and is also used in the discriminator. Upscaling in the generator is performed by nearest neighbor interpolation, while downscaling in the discriminator is performed by convolutions with a stride of 2. The generator is seeded with a 128-dimensional random vector sampled from a standard multivariate Gaussian distribution. Spectral normalization [14] was applied to the generator and the discriminator.

The speckle layer follows the penultimate residual layer of the generator. Here, the feature maps already reached the output image size. Inserting the speckle layer into a deeper part of the network led to poor results. One reason could be that the feature maps in deeper layers have not yet reached the original image size. The speckle layer adds speckle noise with 4 different speckle sizes to all input feature maps, respectively. This means that 8 input feature maps are transformed to 32 output feature maps, whereby 4 feature maps each exhibit the same morphology but with different speckle sizes. These hyperparameters were found by grid-search and stayed the same for all experiments. The input feature maps of the speckle layer are also used to compute channel attention coefficients by applying global sum pooling and two linear layers. The output feature maps of the speckle layer are weighted with these coefficients to filter out unimportant combinations of input feature maps and speckle sizes. A spatial attention approach led to massive checkerboard artifacts and was therefore discarded. The resulting synthetic IVUS images have a size of $256 \times 256$ pixels.

### Dataset

**Fig. 4:**
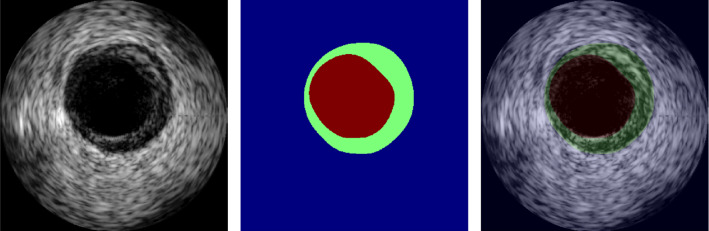
Example image and corresponding mask from the clinical dataset. The image on the right-hand side shows an overlay of image and mask

The underlying IVUS dataset was provided by Balocco et al. [1] and consists of 435 IVUS images captured with a 20 MHz phased array transducer together with corresponding annotated contours marking the lumen border and the media–adventicia interface. The dataset comprises images with calcified and non-calcified plaque as well as bifurcations, side branches and shadow artifacts. The annotated contours were transformed into segmentation masks containing three different classes (lumen, intima/media and adventicia/background). Figure 4 shows an example image with the corresponding segmentation mask.

### Fréchet Inception distance

The Fréchet Inception distance (FID) [10] measures the distance between the generated image data distribution and the real image data distribution by combining mean values and covariance matrices of network activations arising from feeding both image sets into an Inception-v3 model [19], which was pre-trained on the ImageNet dataset [4]. Typically, activations of the penultimate network layer are used to calculate the FID score:

5 $$\begin{aligned} \text {FID} = \Vert \mu _1 - \mu _2\Vert _2^2 + \text {Tr}(C_1 + C_2 - 2(C_1C_2)^{1/2}). \end{aligned}$$

Here, $\mu _1$ and $\mu _2$ are the mean vectors and $C_1$ and $C_2$ the corresponding covariance matrices. Small FID scores and thus small distances between the image data distributions indicate visual similarity of the image sets as well as diversity of the generated image set meaning that mode collapse was prevented. It has not been proven so far that low FID scores induce high image quality when applied to medical images. However, recent works indicate correlation between FID score and realism of generated medical images [13, 21]

### Training

We used the non-saturating GAN loss functions proposed in [6]:

6 $$\begin{aligned} L_D(\mathbf {x}, \mathbf {y}, \mathbf {z})&= - \,\mathbb {E}_{\mathbf {x}\sim p_{\text {data}}} \left[ \log (D(\mathbf {x}\vert \mathbf {y}))\right] - \mathbb {E}_{\mathbf {z}\sim p_{\mathbf {z}}} \left[ \log (1 - D(G(\mathbf {z}\vert \mathbf {y})))\right] , \end{aligned}$$

7 $$\begin{aligned} L_G(\mathbf {y}, \mathbf {z})&= - \,\mathbb {E}_{\mathbf {z}\sim p_{\mathbf {z}}} \left[ \log (D(G(\mathbf {z}\vert \mathbf {y})))\right] , \end{aligned}$$

where $L_D$ and $L_G$ denote the loss functions for discriminator and generator, respectively. Furthermore, $\mathbf {x}$ denotes a real image drawn from the data distribution $p_{\text {data}}$, whereas $\mathbf {y}$ denotes a condition. In this work, $\mathbf {y}$ is a segmentation mask. The random number $\mathbf {z}$ is the input of the generator and is drawn from a standard multivariate Gaussian distribution $p_{\mathbf {z}}$. Finally, *D* and *G* are the discriminator and generator function, respectively.

For defining a baseline GAN, the speckle layer was replaced with an identity mapping (cyan-colored box in the generator sketch of Fig. 3). Everything else remained the same. SpeckleGAN and the baseline GAN were trained with 435, 200, 100 and 50 training examples, respectively. The validation during training was done by means of calculating the FID score between 435 generated images and the whole dataset of 435 real images to make all cases comparable (see “Segmentation evaluation” section for notes regarding overfitting). The GANs were conditioned with the segmentation masks of the dataset to generate synthetic images. This ensures that validation is not affected by artery morphologies, but focuses on textures.

For every combination of model and number of training examples, the best learning rate and learning rate decay scheme was grid searched individually. In summary, the initial learning rates ranged between $1e{-}3$ and $3e{-}4$ and were decreased to $1e{-}4$ or $3e{-}5$ in two steps every few hundred epochs. For optimization we used Adam with $\beta _1 = 0.5$, $\beta _2 = 0.999$ and $\varepsilon = 1e{-}8$. During training, data augmentation was performed by random rotations as well as horizontal and vertical flips. The edge lengths of the square filter windows defining the speckle sizes in the speckle layer were initialized with values ranging from 28 to 48 pixels.

### GAN evaluation

The final evaluation was done by means of calculating the FID score between 1000 generated images and all 435 real images. For generating the synthetic images, the generators were conditioned with artificial segmentation masks produced by superimposing randomly rotated and disturbed ellipses imitating artery lumen and intima/media layers. This approach simulates the way how GANs would be used in practice, namely to augment the dataset they were trained with. As explained in “Fréchet Inception distance” section, the FID-score does not completely ensure reliability when used to evaluate realism of medical image sets. In order to further assess the quality of the synthetic images, we calculated two more metrics: The Jensen–Shannon divergence between gray value distributions of different segmentation classes in ground-truth and synthetic images and the structural similarity (SSIM) index between corresponding ground-truth and synthetic images.

### Segmentation evaluation

The generated IVUS images were used to improve segmentation performances of neural networks with U-Net architecture [16]. The networks consisted of residual blocks [9] in the down- and upsampling path. In each of the three downsampling blocks, the spatial sizes of the feature maps were halved while the numbers of feature maps were doubled up to 256. The upsampling blocks operated vice versa. The input image dimensions were 256$\times$256 and the batch size was 10.

To show that the use of synthetic IVUS data by SpeckleGAN improves segmentation performance when dealing with small datasets, we went through two scenarios:

1. 50 examples available for training a segmentation network,
2. 100 examples available for training a segmentation network.

To get representative performance statistics for the segmentation, we used the remaining examples from the whole dataset (385 for scenario 1 and 335 for scenario 2) as a test set. We used the training sets to train SpeckleGANs and baseline GANs for data augmentation (we did not use the GANs from “GAN evaluation” section). Because of the small datasets in both scenarios, we used the whole training set as a reference set for monitoring the FID score during training and for finally choosing the model which is used to generate the synthetic images for segmentation pre-training. This means that the GANs will tend to overfit on the training set. However, when dealing with extremely small datasets, another split would reduce the amount of data too much in order to get useful results. Furthermore, it is not well studied so far how overfitting via FID scores quantitatively affects GAN performance. In the paper which introduces the FID score [10], the authors also use the training set as a reference set for calculating the FID score.

The best performing GANs each generated 1000 IVUS images by using synthetic segmentation masks as conditional inputs (compare “GAN evaluation” section). The segmentation networks were then pre-trained with the synthetic IVUS data and fine-tuned with the real training data. We used the Dice coefficient and the modified Hausdorff distance [5] to measure the segmentation performances via fivefold cross-validation. The modified Hausdorff distance allows meaningful evaluation of edge alignment for pixel mask-based segmentation results, because it is less sensitive to outliers. The final results were calculated by means of the remaining test sets.

## Results

### Generation of synthetic IVUS images

**Fig. 5:**
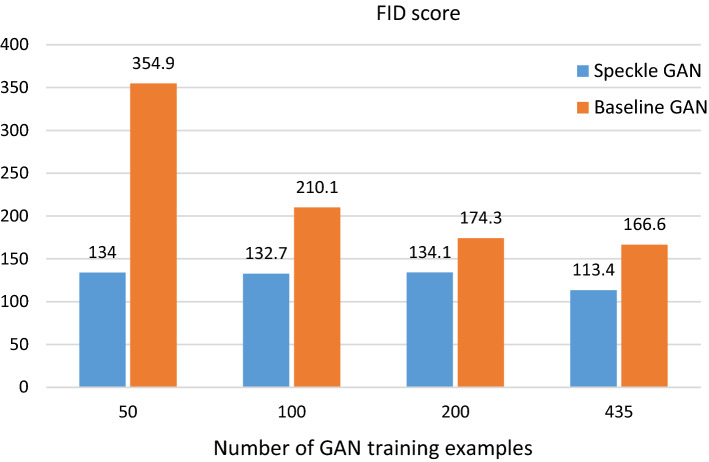
Comparison of SpeckleGAN and the baseline GAN by means of resulting FID scores for different numbers of GAN training examples. A lower values indicates better GAN performance

The chart in Fig. 5 shows the FID scores of image sets generated by SpeckleGAN and the baseline GAN for different numbers of training samples. The image sets generated by SpeckleGAN result in FID scores ranging from 134.0 for 50 training images to 113.4 for 435 training images. The baseline GAN on the other hand reaches values from just 354.9 to 166.6. The performance improvements of SpeckleGAN by means of FID scores for the different numbers of training examples are as follows:

- 50 GAN training examples: 165%
- 100 GAN training examples: 58%
- 200 GAN training examples: 30%
- 435 GAN training examples: 47%

Table 1 shows the GAN performances by means of Jensen–Shannon divergence and SSIM calculated between synthetic and real images. The results are broken down into the number of GAN training examples.

**Table 1:** Jensen–Shannon divergences and structural similarity (SSIM) indices comparing synthetic and ground-truth (g-t) image sets

# Train samples	Datasets	Jensen–Shannon divergence [$10^{-3}$]			SSIM
		Lumen	Intima/media	Adventitia	
50 samples	SpeckleGAN/g-t	**33**.**2**	25.8	**7**.**2**	**0.431** ± ** 0.028**
	Baseline GAN/g-t	289.9	**21**.**1**	55.1	$0.240\pm 0.029$
100 samples	SpeckleGAN/ -t	**7**.**1**	**8**.**1**	8.4	$0.434\pm 0.030$
	Baseline GAN/g-t	28.6	12.0	**4**.**1**	**0.445** ± **0.027**
200 samples	SpeckleGAN/g-t	**4**.**5**	**8**.**7**	**9.3**	**0.440** ± **0.029**
	Baseline GAN/g-t	172.2	11.4	14.5	$0.301\pm 0.027$
435 samples	SpeckleGAN/g-t	**3**.**3**	**3**.**7**	**6**.**2**	**0.443** ± **0.027**
	Baseline GAN/g-t	186.0	10.5	11.7	$0.324\pm 0.025$

Low Jensen–Shannon divergence indicates similar gray value distributions, whereas high SSIM values indicate similar image appearance

**Fig. 6:**
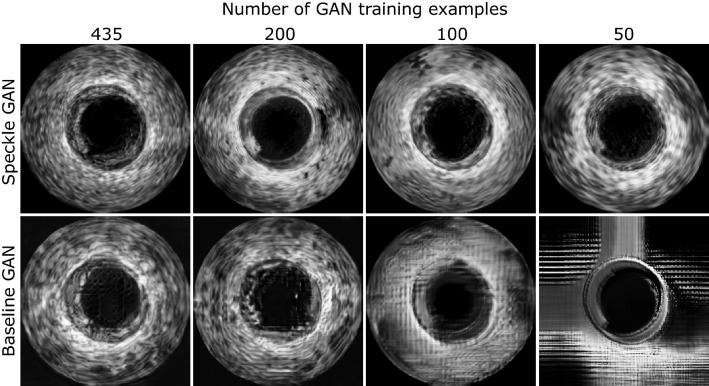
Comparison of IVUS images generated by SpeckleGAN and the baseline GAN for different numbers of GAN training examples. All images were acquired with the same conditional segmentation mask

Figure 6 gives an overview of generated IVUS images for varying numbers of training examples. In all cases, SpeckleGAN generates visually more appealing images than the baseline GAN. The quality of SpeckleGAN images only decreases slightly with fewer training examples, whereas the quality of images generated by the baseline GAN decreases strongly.

### IVUS segmentation

Table 2 shows the segmentation results of both scenarios described in “Segmentation evaluation” section with and without pre-training by means of synthetic images generated by SpeckleGAN and the baseline GAN. The upper table presents the Dice coefficients, whereas the lower table presents the modified Hausdorff distances. We performed t tests in a pairwise fashion to check if the means differ significantly. We note that *p* value correction for multi-hypothesis tests must not be applied in this setting, because we do not perform multiple tests on the same dataset nor do we test one and the same hypothesis on several datasets. The corresponding *p* values are depicted in the four rightmost columns. A low value (typically $p<0.05$) indicates a significant difference in the calculated mean values of the underlying segmentation metrics.

**Table 2:** Comparison of Dice coefficients (upper table) and modified Hausdorff distances (lower table) as a function of the number of training examples

# Samples	Model	Dice coefficient (%)		*p*-values			
				Baseline GAN		No pre-train	
		Intima/media	Lumen	In/Me	Lum	In/Me	Lum
50	SpeckleGAN	$\textbf{83.18} \pm \textbf{0.23}$	$\textbf{93.44} \pm \textbf{0.42}$	$<\,\textbf{0.001}$	0.122	$<\,\textbf{0.001}$	$<\,\textbf{0.001}$
	Baseline GAN	$81.51\pm 92.87$	$92.87\pm 1.18$	$*$	$*$	$<\,\textbf{0.001}$	$<\,\textbf{0.001}$
	No pre-train	$80.02\pm 0.67$	$91.74\pm 1.50$	$*$	$*$	$*$	$*$
100	SpeckleGAN	$\textbf{86.02} \pm \textbf{0.44}$	$\textbf{95.70} \pm \textbf{0.15}$	$\textbf{0.002}$	$<\,\textbf{0.001}$	$<\,\textbf{0.001}$	$<\,\textbf{0.001}$
	Baseline GAN	$85.18\pm 0.46$	$94.95\pm 0.21$	$*$	$*$	0.07	0.495
	No pre-train	$84.79\pm 0.22$	$94.83\pm 0.19$	$*$	$*$	$*$	$*$

# Samples	Model	Mod. Hausdorff dist. [p]		*p*-values			
				Baseline GAN		No pre-train	
		Intima/media	Lumen	In/Me	Lum	In/Me	Lum
50	SpeckleGAN	$\textbf{1.88} \pm \textbf{0.14}$	$3.07\pm 0.40$	0.058	1.000	$<\,\textbf{0.001}$	$\textbf{0.011}$
	Baseline GAN	$2.07\pm 0.30$	$\textbf{3.04} \pm \textbf{1.45}$	$*$	$*$	$<\,\textbf{0.001}$	$\textbf{0.008}$
	No pre-train	$2.54\pm 0.51$	$3.79\pm 2.09$	$*$	$*$	$*$	$*$
100	SpeckleGAN	$\textbf{0.79} \pm \textbf{0.06}$	$\textbf{0.37} \pm \textbf{0.11}$	$\textbf{0.020}$	$<\,\textbf{0.001}$	$\textbf{0.001}$	$<\,\textbf{0.001}$
	Baseline GAN	$0.90\pm 0.09$	$0.92\pm 0.36$	$*$	$*$	0.292	0.071
	No pre-train	$0.95\pm 0.06$	$1.13\pm 0.39$	$*$	$*$	$*$	$*$

The four columns on the right show *p*-values calculated by pairwise *t*-tests. If *p*-values are smaller than 0.05, they are printed boldly

## Discussion

### Generation of synthetic IVUS images

Keeping in mind that the FID score measures the structural similarity of two image sets and their respective diversity, Fig. 5 clearly shows that the baseline GAN fails to generate IVUS image sets with sufficient quality and diversity, if the number of training examples decreases. SpeckleGAN on the other hand hardly suffers from a reduced number of training samples. The images in Fig. 6 show that SpeckleGAN outperforms the baseline GAN for all different numbers of training examples. The visual appearance suffers just slightly from the reduced number of training examples. The baseline GAN generates IVUS images with very blurry and wavy patterns which do not resemble real speckle. For 100 training images, these are smeared out completely and checkerboard artifacts are visible. The baseline GAN completely fails when trained with 50 training samples.

The evaluations by means of the Jensen–Shannon divergence and the SSIM depicted in Table 1 also show the superiority of SpeckleGAN apart from minor exceptions. It is interesting to see, that these exceptions occur in cases, where visual appearance clearly favors the SpeckleGAN results (compare Fig. 6). This can be explained by considering that both metrics do not take into account all image characteristics which are important for IVUS images. For example, a low Jensen–Shannon divergence can be achieved even if the synthetic images do not show speckle at all, because the two-dimensional arrangement of the grey values does not affect gray value histograms. SSIM on the other hand compares luminance, contrast and structure (i.e., the correlation) of two images. In the case of IVUS images luminance and contrast are reliable measures, whereas correlation does not necessarily imply similarity, because of speckle noise. Two almost identical images except from a slight shift of speckle patches can have zero (or even negative) correlation. This also holds for other classic similarity measures such as peak signal to noise ratio (PSNR) or mean squared error (MSE), so these have not been used here.

The authors of [20] used 2075 images of the same clinical IVUS dataset (without segmentation masks) for training a two-stage GAN in order to generate synthetic images. Our approach results in Jensen–Shannon divergences which are one order of magnitude below the values achieved in [20], even for only 100 training examples. In particular, the values obtained for the adventitia layer are far superior, which shows that our approach results in speckle patches leading to gray value distributions resembling the real ones very closely. This could be due to the ability of our algorithm to produce speckles with various sizes over a single image. But also the baseline GAN performs better than the approach in [20] regarding intima/media and adventitia layers when trained with 100 or more samples.

GANs often suffer from mode collapse [17]. This means that only a few or even only a single mode of the data distribution can be generated, which reduces the variety of the samples drastically. SpeckleGAN has the advantage that mode collapse can only affect the morphology (or background) of the image and not the speckle patterns, because these are randomly generated by the speckle layer.

### IVUS segmentation

It has been demonstrated (see Table 2) that pre-training improves the mean Dice coefficient and the mean modified Hausdorff distance regardless of using synthetic images generated by SpeckleGAN or by the baseline GAN. But the improvements due to the baseline GAN are only statistically significant for 50 training examples, not for 100 training examples. In nearly all cases, pre-training with synthetic images of SpeckleGAN leads to better mean segmentation performances than pre-training with images from the baseline GAN. However, the improvement is not statistically significant in three cases of 50 training examples: for the Dice coefficient of the lumen as well as for the modified Hausdorff distance for both intima/media and lumen. It can be seen that pre-training with low quality images from the baseline GAN also improves the resulting Dice coefficients. This indicates that valuable information is even present in the morphology of blurred images.

The evaluation of the Jensen–Shannon divergence in Table 1 and the comparison with [20] shows that the structure of the adventitia in particular benefits from SpeckleGAN. However, its appearance is only of minor importance for the segmentation of lumen and intima/media. The baseline GAN achieves much worse Jensen–Shannon divergences for the lumen. Nevertheless, for 50 training examples the lumen segmentation performance is equivalent or even better by pre-training with images of the Baseline GAN. This leads to the conclusion that realistic speckle does not play an important role for segmentation of the lumen when dealing with 20 MHz IVUS images. Comparing [1, 3, 22] and the results of scenario 2, it can be seen that our approach nearly reached state-of-the-art performance, although our training set was smaller and no special care was taken about optimization of the segmentation network used in this work (see “Segmentation evaluation” section).

## Conclusion

SpeckleGAN improves quality and diversity of generated IVUS images compared to a baseline GAN model without a speckle layer. It generates visually appealing images with defined morphology (conditioned by segmentation masks) even when trained with extremely small datasets of 50 images. SpeckleGAN offers a wide range of possible applications. First of all, it is not limited to generate IVUS images. It could be applied to ultrasound images in general and to other imaging modalities that produce images with speckle such as optical coherence tomography or radar. As seen in the previous section, realistic speckle patterns have only minor impact on the performance when it comes to segmentation of lumen and intima/media layers in IVUS. Classification, detection or tracking tasks which heavily rely on speckle patterns could benefit much more from realistic speckles generated with SpeckleGAN when tackled with data driven algorithms.
